# Evaluating the Impact of the UK's First Day Center Dementia Village: A Qualitative Study

**DOI:** 10.1111/hex.70806

**Published:** 2026-08-11

**Authors:** Megan Rose Readman, Frankii Collins, Julie Rooney, Mark Gabbay, Clarissa Giebel

**Affiliations:** ^1^ Department of Primary Care and Mental Health University of Liverpool Liverpool Merseyside UK; ^2^ National Institute of Health and Care Research Applied Research Collaboration North West Coast Liverpool Merseyside UK; ^3^ Department of Psychology Lancaster University Lancaster Lancashire UK; ^4^ Age UK Wirral Birkenhead Merseyside UK

**Keywords:** day care, day centre, dementia care, dementia village, person centred care

## Abstract

**Background:**

In 2023, Age UK developed the UK's first day centre dementia village at a facility in the North West of England, designed to evoke positive memories and promote engagement in meaningful everyday activities. However, the impacts introducing this dementia village facility have not yet been systematically explored. This study aimed to qualitatively examine the experiences and perceived impacts of engaging with the Age UK dementia village.

**Methods:**

This is an in‐person qualitative semi‐structured interview study with people with dementia (PLWD) and Age UK staff. Eligible participants were adults aged 18 and over who either attended or worked at this Age UK day centre. Data was analysed using reflexive thematic analysis within a social constructivist paradigm.

**Results:**

Twenty‐two interviews comprising 11 members of Age UK Wirral staff and 11 PLWD were conducted. Thematic analysis identified three over‐arching themes: (1) Seeing the client as a person; (2) meaningful activity and engagement; and (3) factors shaping engagement with the dementia village.

**Conclusions:**

Findings suggest that dementia village approaches embedded within day care settings may offer a promising model for enhancing person‐centred care by supporting staff to engage with individuals' identities and tailor meaningful activities. While such initiatives show potential to enhance engagement and social inclusion, their effectiveness is likely to depend on careful adaptation to individual needs, alongside appropriate resourcing and staff support. Further research is required to evaluate long‐term outcomes, cost‐effectiveness, and the ethical implications of increasingly immersive dementia care environments.

## 1 Background

Approximately 57 million people live with dementia globally [1], including almost 1 million people in the United Kingdom (UK) [2]. Supporting people living with dementia (PLWD) to live well remains a central priority, with increasing emphasis on approaches that promote autonomy, social participation, and quality of life rather than focusing solely on symptom management. During the early stages of the condition, unpaid carers, often family members, typically assume primary caregiving responsibilities. However, as care need becomes more complex unpaid carers may be unable to provide the level or intensity of support required [3], often leading to engagement with specialist support services, such as day centres and residential care homes [4, 5]. Despite aiming to support meaningful living, traditional social care settings are often criticised for their clinical and institutional nature which can undermine autonomy and identity and contribute to distress [6, 7, 8].

In partial response to such criticisms, a pioneering care facility, named De Hogeweyk (Dementia Village), was developed in the Netherlands in 2009 [9]. Unlike traditional care homes, Hogeweyk replicates a small village environment, with streets, shops, cafés and gardens, allowing residents to engage in familiar daily activities within a safe setting [9, 10]. The village is structured to support person‐centred care, promoting autonomy, social interaction and meaningful engagement while minimising behavioural challenges commonly associated with institutional care [10].

Opportunities to engage in meaningful activities have consistently been associated with improved wellbeing, quality of life, and maintaining dignity and a sense of self [11, 12]. Dementia villages therefore represent a potentially important shift towards models of dementia care that seek to support personhood through everyday engagement and familiarity. Although empirical evaluation remains limited, emerging evidence suggest that dementia villages support PLWD to maintain engagement with hobbies, routines and aspects of their identity that are often disrupted in traditional care settings [13]. Additional evidence indicates that such environments may improve functioning and reduce reliance on psychotropic medication, with substantial reductions in antipsychotic medication use following implementation of the dementia village model being reported [14]. Existing dementia village models are predominantly residential environments in which people live full‐time within purpose‐built communities. Consequently, evidence regarding their benefits has largely been limited to long‐term residential care settings [13, 14]. Adapting the dementia village concept to a non‐residential day‐centre context may therefore represent an innovative extension of the model, potentially supporting reminiscence, social engagement and community participation while enabling individuals to remain living within the community.

Following the promise of dementia village initiatives, Age UK Wirral, a UK‐based charitable organisation providing support and services for older adults including people with dementia, in collaboration with Challenge Anneka, a British television programme focused on large‐scale community renovation projects, launched an ambitious project in 2023 to transform an unused and unsafe garden space into the UK's first purpose‑built dementia village day centre facility [15]. This dementia village is part of a specialist paid‐for dementia day service attended by individuals with a documented diagnosis of dementia. The village is not a standalone service and users are not separately selected to access it; rather, it forms part of the wider programme of activities delivered during day centre attendance. It is designed to evoke positive memories and encourage engagement in everyday activities within a secure, simulated village setting. The dementia village is laid out like a traditional English village street and includes village ‘shops’, including a record shop, pub, post office, cinema, workshop, bakery and café, filled with items from the 1920s to 1980s. These ‘shops’ are not functioning commercial outlets. Rather, they are staged, exhibit‐like spaces designed to evoke autobiographical memories and stimulate discussion of past experiences. In doing so, the dementia village provides a structured yet familiar environment that is intended to support reminiscence‐based engagement. Consistent with prior evidence [13, 14], Age UK Wirral clients frequently report positive benefits from engagement with the village, including enjoyment, social interaction, and a sense of autonomy.

Reminiscence therapy is a non‐pharmacological intervention in which a therapist uses tangible prompts, such as familiar objects, photos or music, to evoke memories of past experiences and promote discussion of these memories [16]. Prior evidence increasingly supports reminiscence therapy's positive effects on cognitive function, quality of life, depressive symptoms, and communication in people living with dementia [17]. Despite these benefits, Woods et al. [18] observed that any cognitive benefits at the end of treatment were not sustained long‐term. The limited long‐term impact of traditional reminiscence therapy may partly reflect its time‐limited nature and delivery in structured settings, separate from everyday life. By embedding reminiscence into familiar environments, this dementia village offers an opportunity to integrate reminiscence naturally into daily activity, potentially leading to enhanced benefits for PLWD. Despite these insights, the impact of participation in and interaction with such a facility has not yet been systematically evaluated. The present study therefore aimed to (1) explore the experiences of engaging with the dementia village, (2) explore the perceived impacts of engagement, and (3) identify the barriers and facilitators to engagement among service users living with dementia and staff. For this study engagement refers to participation in and interaction with the spaces, and social opportunities provided within the dementia village environment.

## 2 Methods

### 2.1 The Age UK Dementia Village

The Age UK dementia village is a day centre facility laid out like an old English village, featuring a record shop, pub, post office, cinema, workshop, bakery, and café, all filled with items from the 1920s to 1980s.

Operational for 2 years at the time of the study, the Dementia Village forms part of the routine activities offered to attendees of the wider day care service. Individuals are not separately recruited to access the village; rather, all attendees of the service, who have a documented diagnosis of dementia, may engage with the environment during attendance. The service is delivered by professional care staff, who are expected to hold a minimum NVQ Level 2 qualification in Health and Social Care. The facility is part of The North west Coast Living Lab in Ageing and Dementia, where MRR is a linking‐pin researcher supporting co‐developed research and implementation.

### 2.2 Participants and Recruitment

Service users and staff of the day centre were eligible. The organisation delivers support through Early Onset Dementia (EOD) and Elderly Mentally Infirm (EMI) services, and individuals from both units were recruited. Participants were recruited through convenience sampling when the lead author (MRR) was on site. Potential participants received the participant information sheet and, if willing, completed a face‐to‐face interview on the same day. Capacity was assessed prior to participation, and PLWD who lacked capacity to provide informed consent were not included. As all participants were able to provide informed consent independently, families and carers were not involved in recruitment or participation. Ethical approval was obtained from the University of Liverpool [15785].

### 2.3 Data Collection

All interviews were conducted in person, lasted between 10 and 25 min, were conducted by lead author (MR), and were audio‐recorded. The semi‐structured interviews were conducted following two co‐produced topic guides, one for staff and one for clients. Co‐production comprised three steps: (1) developing a draft guide based on literature gaps and study discussions; (2) review by two experts by experience staff recruited from the day centre (FC, JR); and (3) revision following feedback. The final topic guide explored experiences of engaging with the dementia village, either as a member of staff or service user, and barriers and facilitators to service engagement (see Supporting Information: Appendix S1). Participants also completed a brief demographics form, and where relevant provided further information relating to their length of service at the day cenrtre.

### 2.4 Data Analysis

Audio recordings were professionally transcribed verbatim and anonymised. Data were analysed using inductive reflexive thematic analysis informed by Braun and Clarke [19], within a critical realist paradigm by the lead and senior authors (MRR, CG) then reviewed by one public advisor (JR). Analysis followed Braun and Clarkes [20] six step model of thematic analysis: (1) data familiarisation, through repeated reading of transcripts, (2) line‐by‐line manual coding, (3) grouping codes to generate themes and sub‐themes, (4) discussing themes within the wider research team to ensure they accurately represent the data, (5) clearly defining themes and (6) write‐up with supporting quotations from participants. Throughout the analytic process, the lead and senior authors collaborated closely to review codes, categories, and theme development. Any disagreements regarding categorisation or interpretation of the data were resolved through discussion and consensus, with themes refined iteratively to ensure they accurately reflected participants' accounts.

### 2.5 Public Involvement

Two day centre staff formed part of the research team (FC and JR). The experts by experience were involved in conceptualising the study, designing the interview guide, and interpreting the findings. This ensured that the study and the interpretation of findings were grounded in the lived experiences of staff and service users of Age UK Wirral's dementia village.

### 2.6 Reflexivity

Reflexivity was addressed using Guba et al.'s four facets of trustworthiness [21]. Credibility was enhanced through staff expert advisor validation of themes (FC, JR). Transferability was supported by clear descriptions of the setting, recruitment, and interview procedures. Dependability was strengthened through provision of the interview topic guides (see Supporting Information: Appendix S1). Confirmability was supported through ongoing reflexive practice. MRR is a postdoctoral researcher in psychology with experience working with people living with dementia (PLWD). MG is a professor of general practice and Director of the National Institute of Health and Care Research Applied Research Collaboration North West Coast. CG is a senior research fellow with expertise in dementia inequalities. Public advisors (FC, JR) are senior staff at the day centre. The study formed part of The North west Coast Living Lab in Ageing and Dementia, chaired by CG. MRR is embedded at the day centre 1 day per month; participants were known to her but had limited prior contact. MRR kept a reflexive diary and engaged the wider team to strengthen analytic rigour.

## 3 Results

Thematic saturation was deemed to be reached after 22 interviews comprising 11 members of Age UK Wirral staff and 11 PLWD. Participants were aged between 26 and 89 (*M* = 65.54 (SD = 15.29)), with PLWD being older (*M* = 65.55 (SD = 15.29) than staff (*M* = 63.5 (SD = 16.07)). Participants were predominantly female (n = 14, 63.6%), with the proportion of females being slightly higher in staff (72.72%) compared to PLWD (54.54%). All bar one member of staff were currently involved in providing support for clients in the dementia village. The remaining member of staff had previously provided support but was no longer in that role. Staff had been working at Age UK for between 0.5 and 25 years (*M* = 10.5 (SD = 7.43)). A greater proportion of clients living with dementia were part of the EMI service, diagnosed with dementia over the age of 65 (*n* = 7, 63.63%), than the EDO service (*n* = 4, 36.36%), diagnosed with dementia prior to age 65 (see Table 1 for full demographics.).

**Table 1:** Participant demographics.

	Total sample (*n* = 22)	PLWD (*n* = 11)	Staff (*n* = 11)
Age (*M*(SD))	65.54 (15.29)	65.55 (15.29)	63.5 (16.07)
Gender			
Female	*N* = 14	*N* = 6	*N* = 8
Male	*N* = 8	*N* = 5	*N* = 3
Years of experience range; *M*(SD))			0.5–25 years 10.5 (7.43)
Service type			
EMI		*N* = 7	
EOD		*N* = 4	

Abbreviations: EMI, Elderly Mentally Infirm; EOD, Early Onset Dementia; PLWD, people living with dementia; SD, standard deviation.

### 3.1 Qualitative Findings

Three over‐arching themes with various sub‐themes were identified: (1) Seeing the client as a person; (2) meaningful activity and engagement; and (3) Consideration for overcoming potential limiting factors (see Table 2 for a breakdown of themes and subthemes).

**Table 2:** Overarching themes and associated subthemes.

Overarching theme	Subthemes
Seeing the client as a person	Revealing the person through reminiscence
	Using personal knowledge to support person‐centred care
Meaningful activity and engagement	Opportunities for active and purposeful engagement
	Developing a sense of community and belonging
	Flexibility and self‐directed participation
Factors shaping engagement with the dementia village	Practical barriers
	Varied relevance and understanding
	Organisational resources and opportunity

### 3.2 Seeing the Client as a Person

#### 3.2.1 Revealing the Person Through Reminiscence

Participants consistently described the dementia village as stimulating memories through interaction with familiar physical environments and community spaces. Staff described the village as a large‐scale reminiscence tool that helped conversations move beyond pleasantries and care needs towards clients' personal histories, identities, and previous social roles. Engagement with familiar shops and community spaces prompted spontaneous memory recall, recognition, and expression of roles and routines they used to engage in.

okay so the village really is reminiscence on a big scale that's the way I look at it. Especially for the EMI group because they will remember the post office and all of them shops the way you know what they are used to.

(P11, Member of staff)

Staff described how the benefits of reminiscence therapy facilitated by the dementia village appeared to extend beyond the time spent within the village itself. Shared memories and conversations generated within the village continued throughout the day, with staff observing sustained positive affect among clients.

With the village so when the weather's like today if we take people out there they just love it, they are just so enthraled with it a pub and a café and a post office and it's just looking at the smiles on their faces when they go out there and they think they are somewhere else, it's so lovely and those smiles last for a lot longer than the time they are walking around the village.

(P19, Member of staff)

#### 3.2.2 Using Personal Knowledge to Support Person‐Centred Care

Knowledge gained through reminiscence was carried into care practices, shaping how staff approached activities and support. Staff reflected upon how conversations prompted in the dementia village helped them gain a deeper understanding on clients' preferences and personal experiences, supporting more individuals approaches to care.

When they first come, we know bits about their background but not loads. When you've got something to talk about look at physically, they seem to come out their shell a lot more. For example, with the record shop there's songs that trigger them to talk about a lot more about themselves. Or the post office, I've had clients talking about the pram in the post office and talking about child birth and not being able to have a baby so you know so in that way it's been very positive to learn more about them.

(P5, Member of staff)

Staff described how this understanding informed activity planning and day‐to‐day interactions. The village created opportunities to tailor activities around interests, something staff felt was not possible to the same extent when they did not have the tools and prompts afforded by the dementia village.

Theres's people who different walk of life so you've got people who have been bar ladies, they're enjoying the bar. You've got people who really like the music which the reminisce of the music. So, it's a personal need, when people come in the village helps you tailor activities to their needs and preferences. We couldn't really do that, well at least as well, before the village was built.

(P7, Member of staff)

The impacts of this person‐centred approach were also recognised by clients, who noted that tailoring activities to individual preferences facilitated greater social interaction between service users. Clients also recognised the social benefits of activities tailored to individual preferences and interests, particularly where familiar music or activities encouraged interaction and participation from people who would otherwise engage less socially.

When we are playing bowls or whatever, we will have the music on and we will pick different music. There is one guy on our side (EOD service) he doesn't speak a lot, but you put music on, and he will sing along. Which is great because you can get a bit more interaction with him.

(P4, PLWD from EOD service)

Staff additionally described using personally meaningful spaces and stimuli in the dementia village as a tool for emotional regulation and de‐escalation. Familiar environments and music were perceived to help redirect attention and create feelings of calm and comfort during periods of agitation.

But the other day one of the clients became really unsettled and she wanted to go home. I was asked to support her and so I started talking to her about things she likes and she mentioned how she liked music and I thought it would be good idea if I played her favourite songs. So we went out to the record shop and I played some songs for her. She loved it and she started crying against me, but this time it was not because she was sad or unsettled, but because it was happy tears bringing back memories.

(P13, Member of staff)

### 3.3 Meaningful Activity and Engagement

#### 3.3.1 Opportunities for Active and Purposeful Engagement

Participants described a strong desire to use their physical and cognitive skills through activities that felt purposeful and reflected their abilities and interests.

I think I like doing things with other people rather than just sitting around, I think I'm a bit of people person. I think it's good fun to go and watch some old films together in the cinema that's good fun.

(P3, PLWD from EOD service)

Participants described how opportunities for meaningful activity had previously been limited, with the dementia village creating new ways to engage that felt both purposeful and enjoyable. Activities often drew on clients' physical and cognitive skills, reflecting their previous work, hobbies, or interests. This was particularly significant for EOD service users, many of whom had only recently retired and valued opportunities to continue using their abilities in familiar and purposeful ways.

With one of the staff, I do quite a bit of maintenance round the building because we've got a workshop in the village. And a lot of our group because we are under 65 some of them have not long finished work and they don't really want to be sitting round just doing colouring, crosswords, bowls.

(P4, PLWD from EOD service)

Participants also described how the layout of village environment encourages movement in a natural and purposeful way, such as walking between shops and standing to interact with displays, but also in a goal oriented purposeful way. In particular, the outdoor lawn was highlighted as a valuable space that enabled clients to participate in physical activity, such as outdoor bowls, that promotes gentle movement, balance, and coordination. Physical activity was facilitated in combination with social interaction, making movement feel enjoyable rather than structured or clinical.

My favourite is when we are playing bowls and things that like that, I think its keeping my body a little bit you know toned (laughs).

(P3, PLWD from EOD service)

#### 3.3.2 Developing a Sense of Community and Belonging

Engagement in meaningful activities across the village also fostered a sense of community and belonging across service users and staff. Service users described the village as feeling less like a traditional day‐centre facility and more like a social environment in which they could interact and feel recognised and valued as part of a community.

But it's just rather than being like in like a club that cuts yourself off because the shops are there, it's like you are in a good surrounding sort of thing. I feel like it's somewhere that you should be and like belong and things like that.

(P1, PLWD from EOD service)

#### 3.3.3 Flexibility and Self‐Directed Participation

Staff described how the absence of rigid timetabling enabled more spontaneous and person‐centred use of the village, allowing participation to be guided by clients' preferences and energy levels throughout the day, which, supported a sense of autonomy and choice.

Oh gosh its always client initiated you know always, they just pop out whenever they feel is the best for them. Some clients have more energy in the morning so go in the morning other take a while to wake up so may go later its very flexible.

(P19, Member of staff)

Clients also reflected positively on the ability to enter and leave the village freely, describing how this flexibility made engagement feel self‐directed rather than structured or imposed.

You can just go out to the village whenever you want. We just say to the staff we're going out to the village sort of thing and they're okay with that. It's not like they are keeping us to a tight schedule, I like that about it.

(P1, PLWD from EOD service)

### 3.4 Factors Shaping Engagement With the Dementia Village

#### 3.4.1 Practical Barriers

While not preventing engagement entirely, participants described several logistical factors that limited how frequently or effectively the dementia village could be used. As an outdoor facility, weather conditions were perceived to significantly affect accessibility and safety, resulting in engagement being somewhat seasonal and unpredictable.

In the winter it's not used quite as much because it's quite cold. We don't have any heating out there or anything so it's not safe for clients when it's cold.

(P7, member of staff)

The physical size of the village spaces was also perceived as limiting opportunities for group participation and social interaction. Staff reflected that the small size of the “shops” restricted the number of clients who could fit within a shop safely at one time, potentially constraining the wider of sense of community the village aimed to promote.

Personally, I don't think they're big enough there's not much space in there so we can only take a couple at a time and its nicer to do bigger groups, it's much more sociable.

(P21, member of staff)

#### 3.4.2 Varied Relevance and Understanding

Participants also emphasised that the dementia village did not hold the same meaning or appeal for all clients. Some clients from the EOD service described how engagement with the village could become repetitive over time, reducing motivation to frequently engage with the village.

So, I prefer it when you can go and, like, do stuff rather than just looking in and being like, oh, that's nice I remember that. You can only do so much of that after a couple of minutes you know it gets dull.

(P1, PLWD from EOD service)

These accounts suggested that the meaningfulness of the village was influenced by how closely the environment aligned with individuals' own histories and preferences. Some participants reflected that the village's nostalgic design, which largely reflected shops and memorabilia from the 1920s–1980s, resonated more strongly with older generations than with younger clients.

I mean, that the village and the shops they show people, I suppose, in my age, what shops used to be, because that's what shops used to be, when we were kids. But a lot of people here there even are younger than me, and they've not seen their touch in it yet it doesn't show what a shop was like to them.

(P17, PLWD from EMI service)

Staff additionally reflected on how engagement with the dementia village appeared to be influenced by capacity and understanding. While some clients readily engaged with the village, other, often living with more advanced dementia, appeared to be less interested in engaging with the village.

I would say a lot of the people that we have coming in wouldn't find some of it interesting but then we have some that would, it's on capabilities… Some of the clients our side with more advanced dementia just don't seem interested in participating, they would enjoy it I'm sure. But they just don't want to participate, I'm not sure they understand what it is.

(P7, member of staff)

Relatedly, some staff described situations where the realistic nature of the dementia village could contribute to confusion or distress amongst clients who interpret the replica spaces to real functioning shops.

Obviously they see the cake shop and they will be like oh I want a cake and obviously we are trying to explain its just to reminisce but because they look so real they sometimes get confused, very rarely but sometimes some clients do get a bit confused and distressed.

(P5, member of staff)

#### 3.4.3 Organisational Resources and Opportunity

Participants also described how opportunities to engage with the village was shaped by organisational resources. Although the village is intended to be openly accessible, clients noted that supervision was often required, meaning that engagement often depended on staffing ability.

It (usage of the village) it depends really on whether there's somebody there I imagine some of it is locked away and I think you've got to be watched really.

(P08, PLWD from EMI service)

Staff perceived staffing levels as a key factor limiting the extent to which the dementia village could reach its full potential.

The thing that stops you is a staffing problem. They are always dragging staff off to do other things. The benefit (of the dementia village) to the clients is great but it's not reaching its full potential because of the staffing levels.

(P12, Member of staff)

Accounts also highlighted how engagement with the village involved ongoing choices about how clients wished to spend their time within the centre. Some clients described choosing not to use the space due to concerns about missing out on social or group activities taking place inside.

No, I haven't (been into the dementia village) because I think I'm frightened of missing anything going on inside really.

(P15, PLWD from EMI service)

Relatedly, other clients discussed how they were unaware of the opportunity to go out and explore the dementia village whenever they found it appropriate.

I didn't actually know you could just go and look around, I have been waiting to see if someone would take us around it.

(P16, PLWD from EMI service)

## 4 Discussion

This is the first study to qualitatively explore the experiences, perceived impacts, and barriers and facilitators to engaging with a UK day center dementia village initiative. As the facility is now well‐established and built into regular routines for staff and clients, this study was able to reflect on the integration of the facility and its strengths and limitations. Findings highlighted how the dementia village supported person‐centred care by stimulating memories and thus facilitating reminiscence, revealing personal histories and preferences, and enabling staff to tailor activities and interactions to individual needs. Meaningful engagement was not limited to participation in activities alone, but also involved opportunities for autonomy, social connection, purposeful occupation and continued use of familiar skills and roles. Participants experiences challenged the assumption that dementia care is expected to be passive and prescriptive [22], instead demonstrating how supportive environments can enable agency, choice, and active engagement. At the same time, engagement with the village was shaped by contextual factors including personal relevance, cognitive ability, staffing capacity and environmental accessibility.

The dementia village model represents a novel and innovative approach to dementia care delivery [9, 10]. Although empirical evidence remains limited, emerging research corroborates our findings and suggests that this model can overcome barriers associated with traditional care supporting PLWD to remain connected to hobbies, engage in meaningful activity, and, in some cases, improve functioning while reducing reliance on medication [13, 14]. While the Age UK Wirral dementia village was inspired by traditional residential dementia villages, it differs fundamentally in that it is delivered within a day care setting as opposed to a residential long‐term care facility. Despite this difference, our findings align with the limited existing literature. Participants described how the village supports purposeful meaningful activity linked to prior interests and hobbies and fostered a strong sense of community. Importantly, participants valued opportunities to engage in activities perceived as purposeful, familiar and reflective of previous identities, occupations, and abilities. The present findings extend this emerging literature by illustrating how the physical environment itself may actively facilitate person‐centred care through spontaneous reminiscence, self‐directed activity, informal social interaction and opportunities for purposeful occupation within a non‐residential day care context. This suggests that the benefits of the dementia village model may extend beyond long‐term residential care and also be observable in day care environments.

Prior evidence indicates that the benefits from reminiscence therapy, including improvements in cognitive function, quality of life, depressive symptoms, and communication, typically do not last over prolonged periods of time [18]. Notably, in the present study staff discussed how the benefits of reminiscence therapy facilitated by the dementia village appeared to extend beyond the time spent within the environment, with positive affect and engagement persisting throughout the day.

One of the most pervasive barriers to engagement with the dementia village was the risk of boredom or misunderstanding. Some clients, particularly those with younger‐onset dementia, reported that the memorabilia did not reflect their own life experiences and thus prompted less reminiscence. This observation aligns with empirical evidence suggesting that reminiscence therapy is most effective when it incorporates personalised, meaningful cues tailored to an individual's life story, such as bespoke storybooks, photographs, or objects with personal significance, rather than generic or culturally broad materials [23]. The Age UK Wirral dementia village contains highly localised memorabilia, for example, photographs of Liverpool City center and Liverpool and Everton football club memorabilia. Whilst these memorabilia may strongly resonate with long term local clients, they may be less meaningful for individuals who relocated later in life, those from minority ethnic communities whose experiences are underrepresented, or those with different personal interests. Thus, highlighting that engagement with dementia village environments is not experienced uniformly and depends upon how closely the environment aligns with individuals' personal histories, cultural backgrounds, interests and cognitive abilities. Indeed, Chan et al. [24] shows that culturally adapted group‐based activities PLWD lead to significantly higher engagement compared with non‐adapted conventional activities, highlighting the importance of tailoring experiences to participants' cultural and personal backgrounds. Therefore, future initiatives should carefully consider the population that will access their facility alongside the regular rotation and dynamic adaptation of memorabilia and displays to ensure that reminiscence opportunities remain inclusive and responsive to the evolving needs and backgrounds of attendees. However, as we did not quantitatively assess engagement or analyse demographic influences, this assumption remains speculative and requires further empirical corroboration.

Ethical considerations are central to the design and operation of innovative models of care for PLWD. Some argue that the immersiveness of dementia villages may constitute a feigned reality, which risks deception and may conflict with respect for personhood, autonomy and dignity in PLWD [25]. In the present study, staff reported that some clients experienced confusion when interacting with the village's replicated shops, mistaking them for fully functioning commercial spaces. Although the dementia village environment is intended to support reminiscence and engagement, the realism of the settings appears to occasionally provoke distress or frustration. Staff reflections suggest that clear consideration should be given to balancing immersive, meaningful environments with careful supervision, clear communication, and ongoing assessment of individual capacity and responses, to ensure that the therapeutic benefits of the dementia village are realised without inadvertently causing harm. It is, however, important to note that the ethical implications of the dementia village model was not a core focus of the present study. Therefore, future research should systematically explore the ethical implications of dementia villages, including how different designs, levels of realism and individual cognitive capabilities influence the risk of distress, alongside more inclusive cultural contexts and contents.

From an implementation perspective, the Age UK Wirral dementia village represents a promising and potentially scalable model for integration within day care settings, extending existing international dementia village literature which has predominantly focused on long‐term residential care environments. However, this particular dementia village was only achieved through collaboration with *‘Challenge Anneka’* and required substantial resources, making replication without dedicated funding unlikely. Evidence from other innovative dementia care models illustrates that these challenges are not unique to this facility. For example, evidence from Green Care Farms, which provide household‐style, nature‐based care for people living with dementia, demonstrates that successful implementation depends on organisational adaptation, active staff engagement, alignment of care practices with the intended therapeutic environment, and adequate resourcing to sustain meaningful therapeutic benefits [26]. Similarly, participants in the present study described how staffing pressures, limited resources, and wider contextual factors, including accessibility and personal relevance of the environment, influenced opportunities for clients to fully engage with the village. Consistent with implementation frameworks such as the Knowledge‐to‐Action Framework [27], this suggests that successful implementation of dementia villages, and comparable innovative care models, depends not only on their therapeutic potential, but also on adaptation to local contexts, sustainable resourcing, staff support, and alignment with the needs of the populations accessing services. The findings therefore have implications for practice and policy by highlighting the importance of flexible, inclusive, and adequately resourced dementia care environments that support meaningful and self‐directed engagement. Without such structures, the therapeutic opportunities afforded by dementia villages may not be fully realised.

Although this study benefits from being the first exploration of the UK's first day center dementia village, several limitations should be acknowledged. First, the study did not include the perspectives of family members or unpaid carers. Whilst the present study focused specifically on the experiences of clients and staff engaging directly with the dementia village environment, carers play a central role in supporting people living with dementia and may offer important insights into the wider impacts of dementia village engagement on wellbeing and everyday functioning. Future research should therefore incorporate family and carer perspectives to provide a more holistic understanding of the impact of dementia village initiatives. Second, the study recruited only staff and clients from a single day centre located in one of the most deprived areas of the UK. As such, the findings may have limited generalisability to other settings, populations, or service contexts. Third, all interviews were conducted on site, and the study was promoted to staff by public advisors (FC and JR), both of whom hold managerial or senior managerial roles within Age UK Wirral. This may have introduced social desirability bias, with staff potentially withholding critical perspectives. To mitigate this risk, all interviews were conducted by MRR, who sought to create a supportive environment in which participants felt able to share both positive and negative experiences without fear of negative consequences. Additionally, the study was conducted as part of The North west Coast Living Lab in Ageing and Dementia, within which MRR serves as the scientific linking pin for Age UK Wirral. Consequently, all participants were known to MRR prior to data collection. While this prior familiarity may have influenced responses, the study was conducted early in the collaboration, and MRR had met participants on only three to four occasions before participation, with no prior discussion of the study's focus, thereby reducing the likelihood of researcher bias. Finally, whilst the study was informed by collaboration with the day centre's staff, people living with dementia and carers were not directly involved in the design of the study or development of study materials. Greater involvement of people living with dementia and carers in future research may help strengthen the relevance and interpretive depth of dementia village research.

## 5 Conclusion

The day center dementia village represents an innovative approach to dementia care that allows staff to see beyond the diagnosis and engage with clients as individuals with distinct identities. This facilitates person‐centred care, by tailoring activities to individual preferences and fostering meaningful engagement. Engagement in the dementia village enhances social interaction and a sense of belonging among clients. However, participation and efficacy are influenced by factors such as generational relevance of memorabilia, cognitive capacity, and competing priorities. While dementia villages hold promise for normalisation within day care settings, wider implementation depends on sustainable funding and evaluation of long‐term benefits and cost‐effectiveness.

## Author Contributions

**Megan Rose Readman:** conceptualisation, investigation, funding acquisition, writing – original draft, methodology, writing – review and editing, formal analysis, data curation, project administration. **Frankii Collins:** conceptualisation, writing – review and editing. **Julie Rooney:** conceptualisation, writing – review and editing. **Mark Gabbay:** conceptualisation, funding acquisition, writing – review and editing. **Clarissa Giebel:** conceptualization, writing – review and editing, supervision.

## Ethics Statement

This study received favourable ethical approval from The University of Liverpool [15785], and informed consent was obtained from all participants.

## Conflicts of Interest

The authors declare no conflicts of interest.

## Patient and Public Involvement

Two Age UK staff from this facility served as public advisor researchers, co‐designing the interview guide and supporting interpretation of findings.

## Supporting information


Supporting File

## Data Availability

The data that support the findings of this study are available on request from the corresponding author. The data are not publicly available due to privacy or ethical restrictions.
