# Supplementary material for: Evaluating the Impact of the UK's First Day Center Dementia Village: A Qualitative Study
Source: Health Expect. 2026 Aug 11;29(4):e70806. doi: 10.1111/hex.70806 (PMC13459194; doi:10.1111/hex.70806)
Supplement: Supplementary file 1 [file HEX-29-e70806-s001.docx]

**Appendix**

**Interview topic guide for people Age UK Wirral Staff**

*Note: use separate topic guide for each interview.*

**Interview #:______________**

**Pre-interview checklist for interviewer:**

**☐** Participant information sheet reviewed by interviewee

**☐** Consent process completed

**Participant information**

**Introduction**

Thank you for agreeing to take part in an interview for our study today. This interview is expected to take up to a maximum of 30 minutes. Is that OK with you, and do you need to finish earlier for any reason?

[*Interviewer introduce themselves. Include job title, research interests, gender so we both know how to address each other]*

Today we will discuss the Age UK Wirral dementia village and we want to understand your experience of engaging with the village.

Do you have any questions before we start? I will now switch on the audio recorder.

[SWITCH ON AUDIO RECORDER]

*Discussion begins (below), make sure to give people time to think before answering the questions and don’t move too quickly. Use the probes to make sure that all issues are addressed, but move on when you feel you are starting to hear repetitive information and allow for flexibility in the direction that the participant takes the conversation.*

| **Check** | **#** | **Question** | **Probe** |
| --- | --- | --- | --- |
| **Introduction – Understanding the individual participating** | | | |
| ☐ | 1 | To start with can you please tell me a little bit about yourself and your role at Age UK Wirral day centre? | -How long have you been working with people living with dementia?  -What does your day to day look like at Age UK Wirral?  -Do you have any formal health care training? |
| **The dementia village** | | | |
| ☐ | 2 | Can you tell me about your experience with the dementia village? | -How often do you engage with the dementia village? |
| ☐ | 3 | Can you tell me a bit more about how you support clients within the dementia village? | -Typically, how would a client go about accessing the village?  -How are you expected to support clients to use the village? |
| ☐ | 4 | What’s your impression of how clients use and engage with the dementia village, and are there any specific aspects of the dementia village that clients seem to particularly like or have difficulty with? | - Do clients enjoy their time in the dementia village?  - Is there anything in particular that is well liked? |
| ☐ | 5 | Have you faced any barriers in client engagement with the village? | - Do clients ever struggle to engage with the village? |
| ☐ | 6 | In what ways could the dementia village be improved and/or further developed? | - Is there anything you would like to see changed to support clients? |
| **Impact of the dementia village** | | | |
| ☐ | 6 | In what ways, if any has using the dementia village impacted the clients at Age UK Wirral day centre and if so how? |  |
| **Concluding remarks** | | | |
| ☐ | 7 | Lastly, is there anything else you would like to add that we have not touched upon yet? |  |
| Thank you for your time. I’m going to switch off the recorder now. [SWITCH OFF AUDIO RECORDER] | | | |
| **Data collection diary** | | | |
| *Add anonymous comments only, to aid interpretation of transcripts* | | | |

**Interview topic guide for people with dementia**

*Note: use separate topic guide for each interview.*

**Interview #:______________**

**Pre-interview checklist for interviewer:**

**☐** Participant information sheet reviewed by interviewee

**☐** Consent process completed

**Participant information**

**Introduction**

Thank you for agreeing to take part in an interview for our study today. This interview is expected to take up to a maximum of 30 minutes. Is that OK with you, and do you need to finish earlier for any reason?

[*Interviewer introduces themselves. Include job title, research interests, gender so we both know how to address each other]*

Today we will discuss the Age UK Wirral dementia village and we want to understand your experience of engaging with the village.

Do you have any questions before we start? I will now switch on the audio recorder.

[SWITCH ON AUDIO RECORDER]

*Discussion begins (below), make sure to give people time to think before answering the questions and don’t move too quickly. Use the probes to make sure that all issues are addressed, but move on when you feel you are starting to hear repetitive information and allow for flexibility in the direction that the participant takes the conversation.*

| **Check** | **#** | **Question** | **Probe** |
| --- | --- | --- | --- |
| **Introduction – Understanding the individual participating** | | | |
| ☐ | 1 | To start with can you please tell me a little bit about yourself and your diagnosis of dementia? | -How old were you when you were diagnosed with dementia? |
| ☐ | 2 | Can you tell me about your experiences at the Age UK Wirral day centre? | -How long have you been coming to the Age UK Wirral day centre? |
| **The dementia village** | | | |
| ☐ | 3 | Can you tell me about your experience specifically with the dementia village? | -How often do you use the dementia village?  -Which shops do you use the most? |
| ☐ | 4 | When you engage with the village, are you on your own or do you go with a member of staff or other service user? How easy is it to access the village on a daily basis? |  |
| ☐ | 5 | Is there anything in particular that you like about the dementia village? | - Do you enjoy using the dementia village?  - What do you like most about the village? |
| ☐ | 6 | Is there anything you feel that could be improved about the dementia village? | -Is there anything you dislike about the dementia village?  - Is there anything you would like to see changed? |
| **Impact of the dementia village** | | | |
| ☐ | 7 | Do you feel that using the dementia village has any impact on you, if so, how? | - |
| **Concluding remarks** | | | |
| ☐ | 8 | Lastly, is there anything else you would like to add that we have not touched upon yet? |  |
| Thank you for your time. I’m going to switch off the recorder now. [SWITCH OFF AUDIO RECORDER] | | | |
| **Data collection diary** | | | |
| *Add anonymous comments only, to aid interpretation of transcripts* | | | |
